# Estimating spatio-temporal distributions of mosquito breeding pools in irrigated agricultural schemes: a case study at the Bwanje Valley Irrigation Scheme

**DOI:** 10.1186/s12936-020-3113-3

**Published:** 2020-01-22

**Authors:** April N. Frake, Willy Namaona, Edward D. Walker, Joseph P. Messina

**Affiliations:** 1 Department of Geography, The University of Alabama, Tuscaloosa, AL 35487 USA; 2 Agricultural Engineering Department, Lilongwe University of Agriculture and Natural Resources, Bunda College, P.O. Box 219, Lilongwe, Malawi; 3 Department of Microbiology and Molecular Genetics, Michigan State University, East Lansing, MI 48823 USA; 4 College of Arts and Sciences, The University of Alabama, Tuscaloosa, AL 35487 USA

**Keywords:** Malaria, Spatio-temporal modeling, Irrigation

## Abstract

**Background:**

The association between irrigation and the proliferation of adult mosquitoes including malaria vectors is well known; however, irrigation schemes are treated as homogenous spatio-temporal units, with little consideration for how larval breeding varies across space and time. The objective of this study was to estimate the spatio-temporal distribution of pools of water facilitating breeding at the Bwanje Valley Irrigation Scheme (BVIS) in Malawi, Africa as a function of environmental and anthropogenic characteristics.

**Methods:**

Irrigation structure and land cover were quantified during the dry and rainy seasons of 2016 and 2017, respectively. These data were combined with soil type, irrigation scheduling, drainage, and maintenance to model suitability for mosquito breeding across the landscape under three scenarios: rainy season, dry season with limited water resources, and a dry season with abundant water resources.

**Results:**

Results demonstrate seasonal, asymmetrical breeding potential and areas of maximum breeding potential as a function of environmental characteristics and anthropogenic influence in each scenario. The highest percentage of suitable area for breeding occurs during the rainy season; however, findings show that it is not merely the amount of water in an irrigated landscape, but the management of water resources that determines the aggregation of water bodies. In each scenario, timing and direction of irrigation along with inefficient drainage render the westernmost portion of BVIS the area of highest breeding opportunity, which expands and contracts seasonally in response to water resource availability and management decisions.

**Conclusions:**

Changes in the geography of breeding potential across irrigated spaces can have profound effects on the distribution of malaria risk for those living in close proximity to irrigated agricultural schemes. The methods presented are generalizable across geographies for estimating spatio-temporal distributions of breeding risk for mosquitoes in irrigated schemes, presenting an opportunity for greater geographically targeted strategies for management.

## Background

Irrigation is a prominent tool for ensuring food security through enhancing agricultural productivity [1, 2] and is often discussed in relationship to the health and economic benefits for smallholder farmers, or more generally, developing countries. Yet, irrigation has profound, often complex, effects across domains. Irrigation has the potential to increase crop production [3] and diversification [4] and has been shown to improve health outcomes through dietary diversification [4]. However, agrarian transformation of the landscape for irrigation is also associated with altering biotic interactions within ecosystems that encourage vector and pathogen transmission, including malaria [5–8]. Further, increased erosion, salinization, deterioration of water quality, proliferation of aquatic weeds, and eutrophication are associated with the expansion of irrigated agriculture [9, 10].

The capacity for irrigation development across sub-Saharan Africa (SSA) is considerable, with only 14% of irrigation potential developed across the region [11]. The drivers of irrigation underdevelopment in the SSA region according to Ward et al. [11] are fourfold: (1) economic incentives for investment have historically been limited; (2) a lack of tradition of irrigated agriculture throughout the region; (3) the remote location of many sites often makes irrigation development cost prohibitive; and (4) a history of disappointing development results during the 1970s. Yet, despite a period of reticence to further expand irrigation by governments and donors, recent years have brought a renewed interest in irrigation potential, particularly in light of food insecurity concerns [11].

Malawi is a landlocked country in southeastern Africa where 80% of the population are smallholder farmers [12] highly dependent on rainfed production of subsistence crops. Concentrated during the months of December–March [13], Malawi’s rainy season is distinct and unimodal [14]. Given the widespread dependence on rainfed agriculture, seasonal weather fluctuations considerably impact Malawi’s agricultural production, prompting promotion of irrigation interventions to not only mitigate food insecurity concerns, but also as a means of poverty reduction and economic growth [15]. Irrigation development in Malawi dates back to the 1940s with the construction of the Limphasa irrigation scheme in the Nkahata Bay District [16]. Between 2006 and 2014 alone, irrigated land for smallholder farming increased by almost 300% [17]. By 2015, the area under irrigation in Malawi had expanded to 104,298 hectares (ha) across 1703 schemes [15]; over 96% of irrigation schemes are categorized as ‘smallholder’ operating predominately by gravity-fed (57%) or treadle pump (29%) irrigation. Other irrigation technologies include watering cans (7%) and motorized pumping systems (7%) [15].

Adopted in 2016, the National Irrigation Policy (NIP) [18] works to address critical issues affecting the irrigation sector in an effort to achieve the Government of Malawi’s (GoM) vision of achieving a total irrigated area of 220,000 ha by 2035 [15]. Working to achieve the aspirations of the NIP is the Green Belt Initiative (GBI), aimed at increasing production and productivity of crops along with sustained economic growth. Under the GBI, the Malawi government has offered local and foreign investors 1 million hectares of land for irrigation development [19]. As irrigation continues to be developed across Malawi and the SSA region, accurate and timely information describing the impacts of irrigation development and rehabilitation of existing schemes for ecosystem services is imperative. Further, given the relationship between irrigated agriculture and infectious disease (e.g., [20–23]), it is prudent to consider how infrastructure and land use and land cover change (LULCC) for irrigated agriculture may alter disease systems.

Malaria is of considerable public health importance to Malawi. In 2017, 4.9 million confirmed cases were reported to the World Health Organization; 3613 resulted in death [24]. Malaria is transmitted via the bite of an infected female *Anopheles* mosquito and surface water is critical for mosquito survival—the first three stages of a mosquito’s life cycle are aquatic. As surface water for irrigation expands across space, so too does the potential for mosquito breeding pool formation and persistence. Malaria transmission is often seasonal, peaking during or immediately after the rainy season [25]. Given the availability of surface water for irrigation, irrespective of seasonal rainfall, irrigated agriculture has the potential to significantly change malaria disease dynamics through increased availability of breeding sites and lengthening the seasonal transmission window.

The association between irrigation and mosquito production is well documented in the literature (see e.g. [21, 26]). However, irrigated schemes are treated as homogenous spatio-temporal units with little consideration for how breeding potential varies across space and time. Irrigated schemes may change seasonally in crop production and distribution and not every irrigation scheme receives sufficient water resources to operate on an annual, but rather only seasonal basis. In addition, a proportion of some land cover types found in irrigation schemes are not agricultural, but rather engineered structures including concrete canal networks. The heterogeneity of irrigation scheme’s spatio-temporal distribution results in asymmetrical breeding risk. The spatial structure of heterogeneity is the product of environmental and anthropogenic factors, including, soil type, timing and intensity of irrigation, drainage, and crops type(s) being cultivated. Each of these factors, independently, and in combination, influence the amount and duration of pooled surface water available to mosquitoes for breeding. Here, the results of a characterization study at the Bwanje Valley Irrigation Scheme (BVIS) are reported to explore the influence of the scheme’s spatial distribution on breeding pool formation. Environmental characteristics and anthropogenic influences pertinent to breeding distribution are assessed; these data are used to generate three spatio-temporal breeding scenarios across the scheme. Results illustrate how perturbations to irrigated systems in the form of water availability, water management, and crop cover influence the distribution of aggregated water bodies and thereby influence disease ecology for the local area.

## Methods

### Study site

**Fig. 1:**
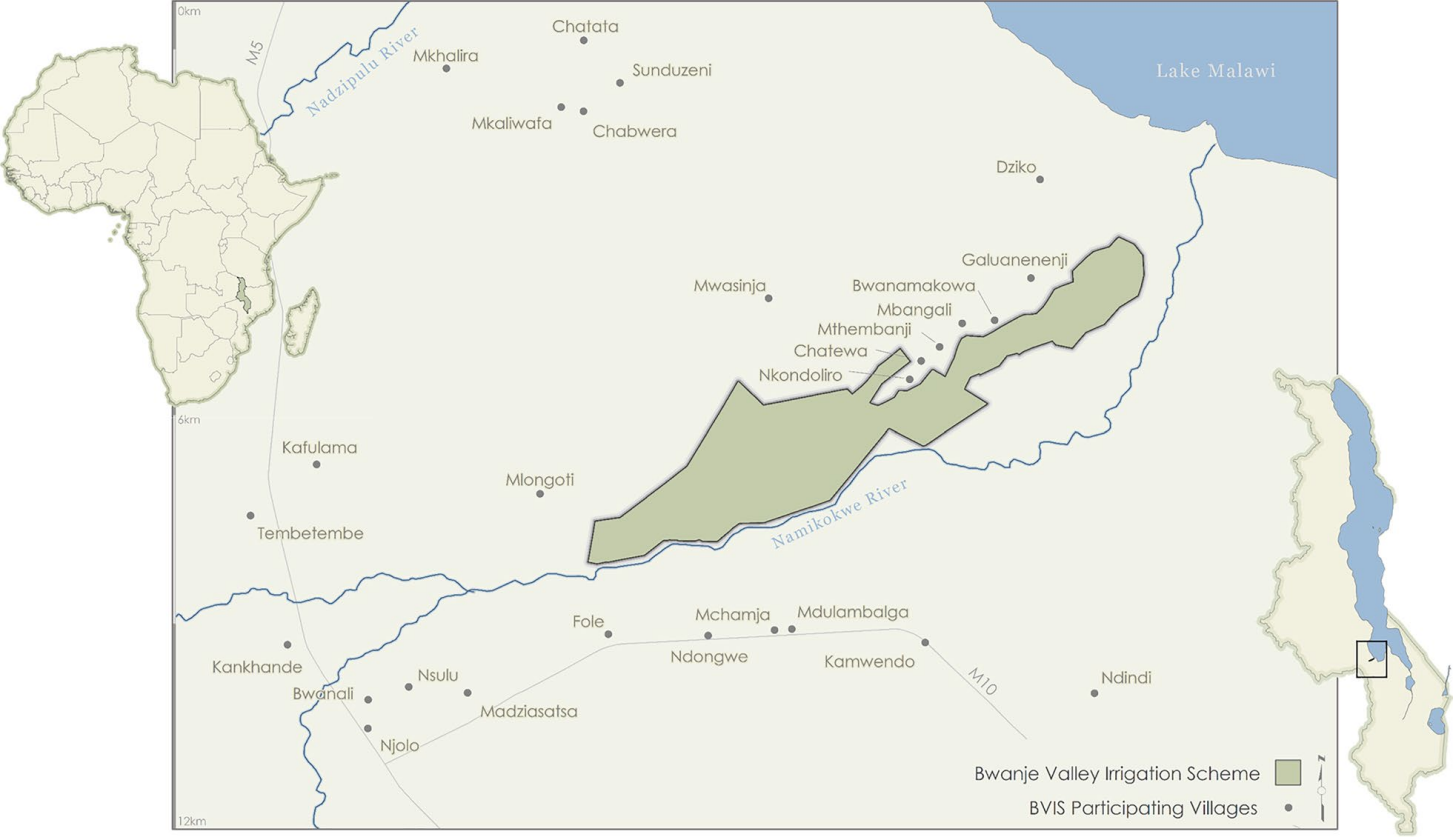
The Bwanje Valley Irrigation Scheme (BVIS) located in Dedza district, Malawi

BVIS is an example of a typical smallholder irrigation scheme in Malawi (Fig. 1). The scheme was established in 2000 through cooperation between the Malawi and Japanese governments with the intention of improving household food security and incomes [27]. Grant aid of $15 million USD was provided by Japan, mediated through the Japanese International Cooperation Agency (JICA) [28]. BVIS is a gravity-fed scheme located in the Shire watershed of central Malawi, where annual rainfall is approximately 867 mm and temperatures range from 17.2 to 27.3 °C [29]. Spanning 800 ha, the scheme diverts water from the Namikokwe River (a.k.a., Nankhokwe River) to ~ 30 × 30 m agricultural plots via a series of concrete main and branching canals, and earthen tertiary canals. During the 2016 growing season, 2067 farmers participated at BVIS from 14 surrounding villages; 1089 farmers were women [M. Tarsizio, pers. commun., 2016]. Veldwisch et al. [28] provide a comprehensive commentary on BVIS including the schemes’ historical development and subsequent ‘travails.’

### Study design

**Fig. 2:**
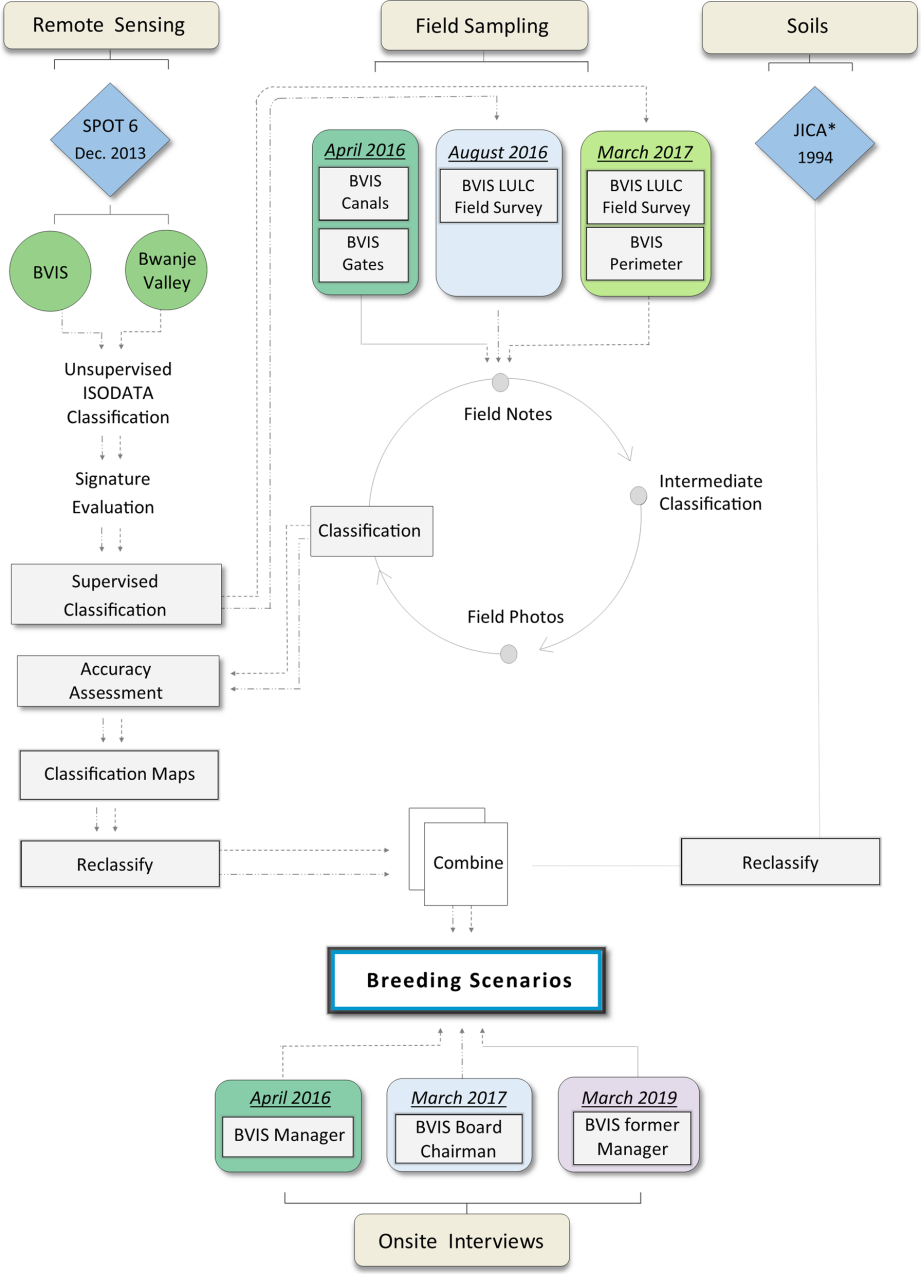
Conceptual framework for the characterization of BVIS

Four sources of information are used to characterize BVIS: (1) Satellite imagery from the SPOT-6 multispectral sensor at two time periods; (2) Spatial structure and land cover information derived through field sampling during rainy and dry seasons; (3) Soils data taken from [30]; and (4) Onsite interviews with BVIS scheme personnel conducted in 2016, 2017, and 2019. The 2016 interview was conducted with the scheme manager, who was often present during field surveys to provide insight and commentary. In 2017, an interview with the BVIS board chairman was held in the absence of a permanent scheme manager. The 2019 interview was conducted with the former BVIS manager interviewed in 2016. Survey instruments are available at [31–33]. The conceptual framework for this study is presented in Fig. 2.

### Environmental characteristics

#### Soils

**Fig. 3:**
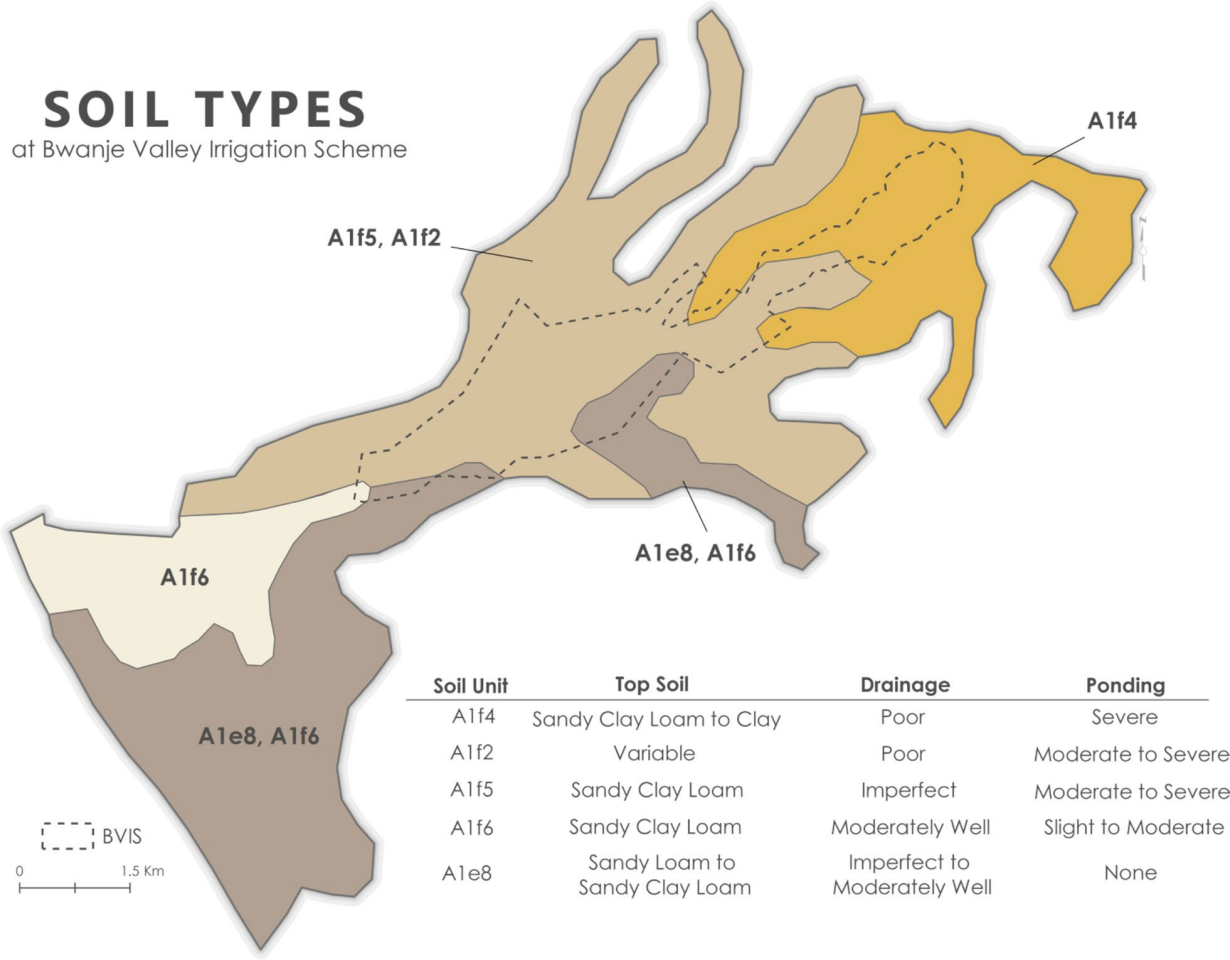
Soil types at BVIS. Data and soil characteristics are taken from JICA [30]

The BVIS is situated across four soil unit areas made up of five primary soils whose parent materials are fluvial, colluvial, and/or lacustrine sediments; two soil units contain more than one soil type as described by [30] (Fig. 3). Top soil (0–30 cm) types are sandy loam, sand clay loam, and sandy clay loam to clay. A notable consideration for this study is the drainage capacity for each soil. The dominant soil units at BVIS (A1f5, A1f2, A1f4) are typified by poor to imperfect drainage with moderate to severe ponding potential.

#### Land cover

Multispectral SPOT-6 images of the study area were acquired on December 04, 2013 and April 1, 2014 at a spatial resolution of 6.0-m with four spectral bands. Scenes were selected for analysis to reflect distinctions in dry and rainy season cropping patterns at BVIS, respectively. Data preparation steps included geometric ortho-correction to ensure positional accuracy using ground control points collected prior to analysis and a 30-m Digital Elevation Model (DEM) from Shuttle Radar Topography Mission (SRTM) [34]. In addition, sensor calibration, or conversion of digital numbers (DNs) back to radiance, followed by a solar correction to top-of-atmosphere reflectance was performed.

Image classification was carried out using an unsupervised ISODATA classification where 255 classes were selected at 0.98 convergence (see e.g., [35]). Signature evaluation was conducted using the Transformed Divergence measure with separability markers of > 1975 deemed acceptable. Using the edited signature set, a Maximum Likelihood supervised classification was performed. Data preparation and image classifications were processed in ERDAS IMAGINE™ 2014.

Field sample sites were selected by stratified random sampling of land cover classes from the supervised classification for each image: 11 classes in the dry season and 9 in the rainy season. A total of 242 points were selected during the dry season and 72 during the wet season. Fewer sample sites were selected during the rainy season given that there were fewer classes to sample and previous fieldwork had shown that rice was the predominant crop across the scheme.

Field based surveys of land use and land cover (LULC) were conducted during the 2016 dry season and 2017 rainy season. A total of 235 dry season samples were collected over an 8-day period in mid-August 2016 by a field team of three researchers. These samples included 185 of the sites selected from the random stratified sample, and 50 additional ‘accuracy assessment’ points collected to further assist with classification assessment. Rainy season samples included 68 training sites and 36 accuracy assessment sites sampled over a 9-day period in early April 2017 by a field team of two researchers. At each site, the location and elevation, along with geotagged photographs were captured using a handheld Garmin™ Monterra Global Positioning System (GPS) unit. In addition, field notes describing the LULC at each sample site along with descriptions of LULC in all directions were recorded. Where land cover of surrounding areas differed from that recorded at the sample site, the direction of these areas along with approximate distances were included within field notes. Field note transcriptions and associated GPS data collected at each sample site were combined for classification analysis.

Classification of LULC followed a two-step process: (1) Sites were categorized by land use (agricultural land), land cover type (active or fallow), and features (e.g. maize, rice, bare earth); (2) Field notes were used to include information on stage of agricultural growth, appearance of soil, density of plantings, presence of water within irrigation canals, and locations of trees relative to agricultural growth for each feature identified in step 1. Point shapefiles of all sample sites, by season, were generated in ArcMap™ 10.5.1, then overlain with supervised classification imagery.

**Fig. 4:**
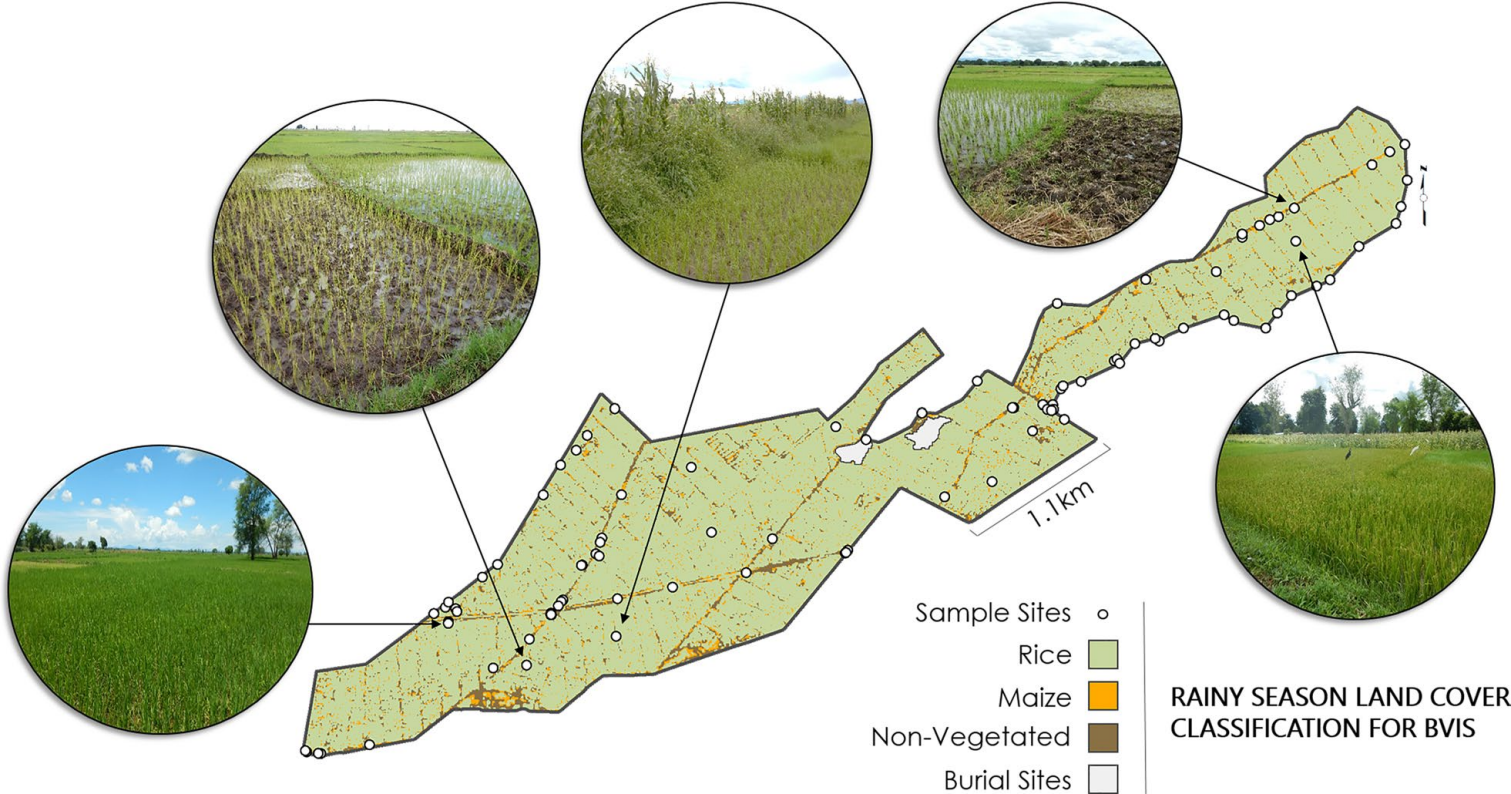
Rainy season land cover classification for BVIS including field photos of varied stages of rice growth throughout the scheme

Maximum Likelihood classification for the rainy season yielded 9 classes of land cover, of which seven were classified as ‘Rice’, one as ‘Maize’ and one as ‘Non-Agricultural’ (Fig. 4). The number of supervised classes assigned as ‘Rice’ is attributable to the variations in growth stages observed during sampling. While rice plants in some plots were in panicle formation stage, other plants had begun flowering, or were mature. Rice on other plots had been harvested, releasing the plots to renewed planting. The Non-Agricultural class included irrigation canals, concrete and earthen, along with roads and pathways.

One particular challenge of the rainy season classification was presence of maize grown in single or double rows, in the narrow spaces immediately adjacent to irrigation canals. These areas are situated at a slightly higher elevation than the plots (~2-ft), owing to the gravity-fed design of the scheme. The proximity of maize in relationship to the structures within the ‘Non-Agricultural’ class is the cause for the mixing of pixels observed in areas immediately adjacent to roadways and irrigation canals.

The combination of water availability from seasonal rains and the Namikokwe River during the rainy season allows for the dominant cultivation of rice at BVIS, occurring across 87.9% of the scheme. The primary rice varieties are kilombero, a long-grain aromatic rice, and faya [M. Mafosha, pers. commun., 2019]. BVIS personnel direct farmers to begin planting in mid-December, with harvesting around mid-February [M. Mafosha, pers. commun., 2016]. However, the varied stages of rice growth observed during land cover analysis from this study suggests that farmers are able to practice governance over their plots cropping timeline. Besides growing in areas immediately adjacent to irrigation canals, two concentrated areas of maize are cultivated during the rainy season along the southern boundary of the scheme. Rehabilitation plans for BVIS were prepared in 2005 by JICA and included fine leveling for plots [28, 36]. However, according to interviews with BVIS management, the plots in these areas were not leveled as well as in other areas. As a result, these areas are situated at a slightly higher elevation than those immediately surrounding them, preventing efficient water flow and cultivation of rice [M. Mafosha, pers. commun., 2019].

Lack of adequate water resources in the dry season limits the amount and distribution of cultivation at BVIS. To assist in differentiating between active and fallow vegetation in the dry season, the Normalized Difference Vegetation Index (NDVI) was used to assess agricultural growth. The NDVI index is sensitive to live, green plants, elucidating the difference between the near-infrared and visible red owing to chlorophyll’s absorption of visible light, and the cell structure of leaves effect on the reflectance of near-infrared light [37]. Of the 207 sample sites assessed across BVIS, active agriculture were sampled at 46. These 46 points were combined with the NDVI scene, their range of values (0.14 to 0.48) analysed, then visually inspected for variations of NDVI in relation to the supervised land cover classes. This analysis in combination with consideration of feature descriptions of each sample point determined class assignments of LULC.

**Fig. 5:**
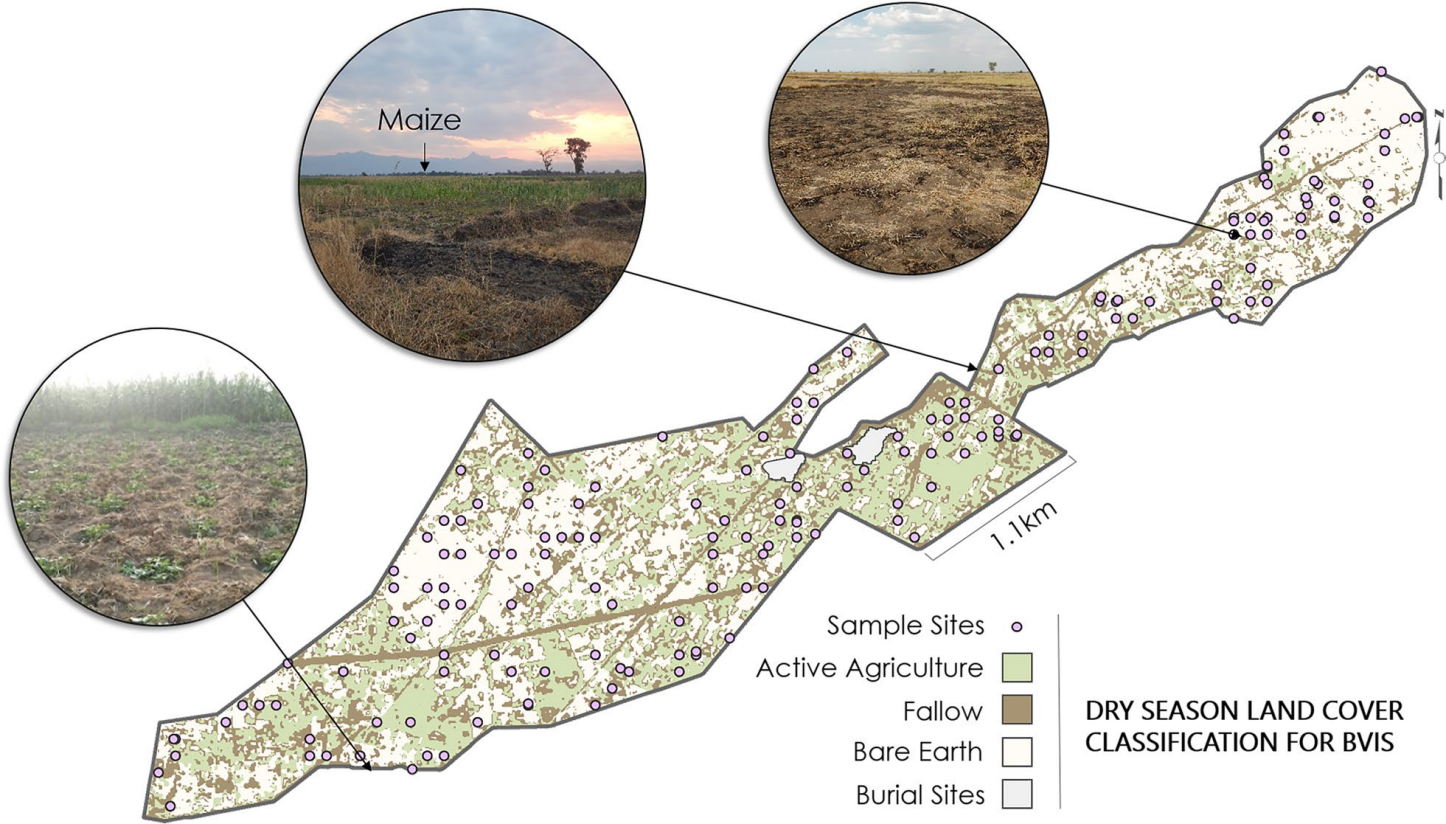
Dry season land cover classification for BVIS including field photos of varied land covers surveyed

Developing a classification system for the dry season presented a number of challenges: (1) Cultivation was widely dispersed throughout the scheme; (2) Bare fields spectrally resembled earthen roadways and tertiary canals; and (3) Maize and unmanaged grasses were observed growing immediately adjacent to and often overhanging irrigation canals. The maximum likelihood classification analysis showed eleven significant classes of land cover of which five were classified as ‘Fallow’, four as ‘Non-Vegetated’, and two as ‘Active Agriculture’ (Fig. 5).

Dry season land cover was also more diverse than initially expected. The appearance of fallow fields varied significantly, attributable to farmer decision-making on the method of field clearing: burning, hand weeding, or no clearing. The ‘Fallow’ land cover category included observations of ‘Charred Ground’ from burning at the completion of cultivation, dried fields were predominately characterized by dry, harvested rice, and shrubbery—a term used to describe various forms unmanaged grasses and weeds. The ‘Non-Vegetated’ class is typified by irrigation structure’s including dirt roadways, concrete and earthen canals. However, pixels in this class are also located within agricultural fields. These mixed pixel effects are attributable to the complexity of mapping *land use* versus *land cover* in an irrigation scheme. In this case, even within an over-arching ‘Agricultural Land’ land use classification, a portion of the land functions as agricultural plots while the remainder serves as infrastructure for agricultural growth. Dirt roadways and earthen canals at BVIS are constructed from native soils. Thus, spectrally, they resemble bare fields where farmers have cleared their plots post-harvest.

The ‘Active Agriculture’ class included observations of ‘Maize,’ ‘Beans,’ ‘Cowpea’ and in rare cases, intercropping of maize and beans. The majority of observations of maize sampled were mature (72%). Whereas observations of bean and cowpea growth stages were evenly distributed from young to mature.

**Fig. 6:**
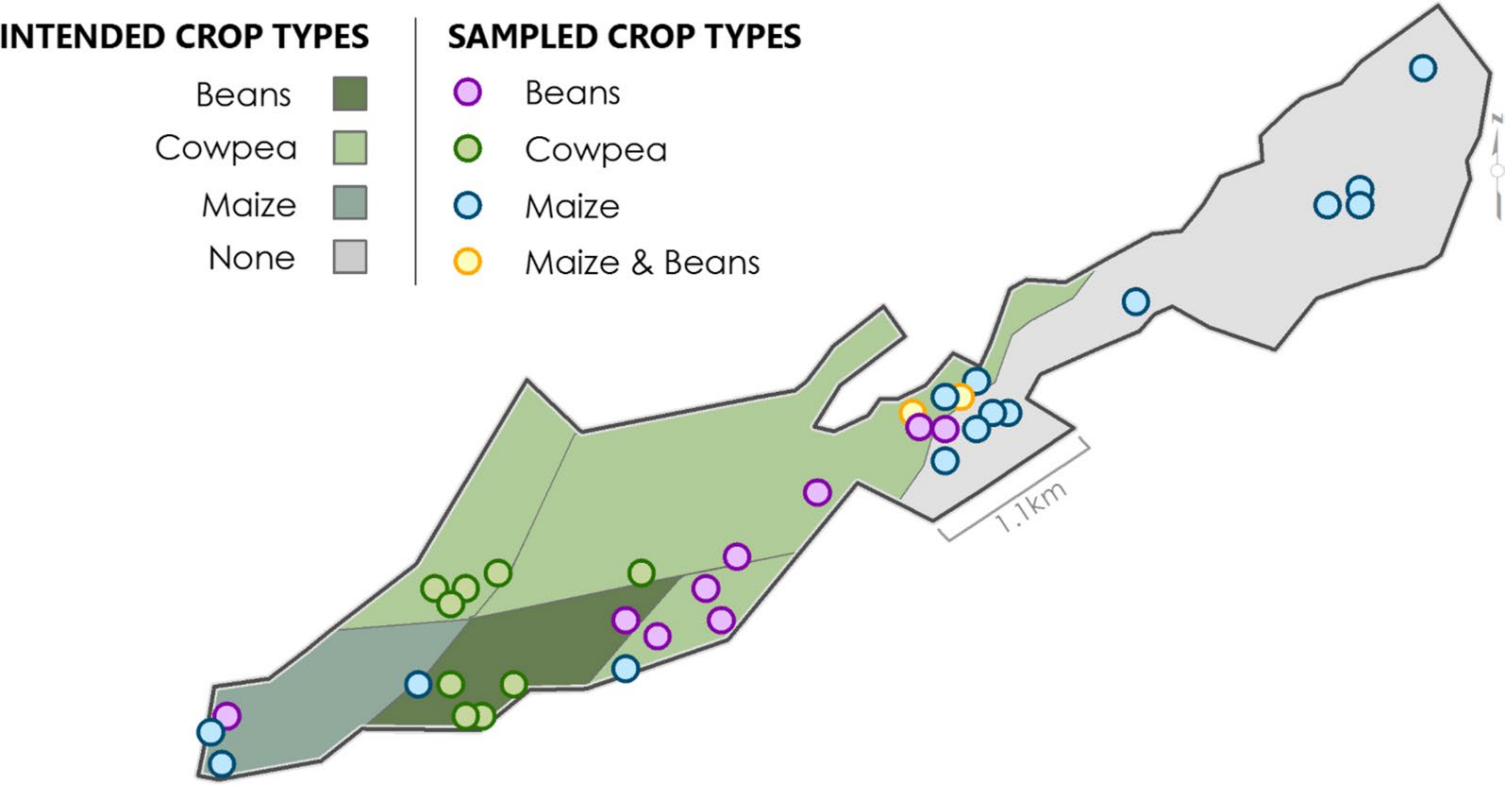
Variation between BVIS Farmer Cooperative's intended distribution of crop types in relationship to where crops were sampled during field surveys

The extent of dry season cultivation at BVIS is determined by water availability; typical cultivation occurs over only 300 hectares (ha) [M. Tarsizio, pers. commun., 2016]. The crop types and hectarage cultivated during typical dry seasons are: Maize (200–250 ha), Beans (50–60 ha), Cowpea (5–10 ha), and Soya (50 ha) [M. Tarsizio, pers. commun., 2016]. Management reported that water scarcity in 2016 limited cultivation to 180 ha: 50 ha were allocated to maize, 120 ha to cowpea, and 10 ha to beans. The decision to forego growing soya and expand cowpea cultivation during the 2016 season was in an effort to mitigate the impacts of drought [M. Tarsizio, pers. commun., 2016]. The intended areas of the scheme for cultivation of cowpea, maize, and beans by scheme management for the 2016 dry season are presented in Fig. 6. However, field sampling demonstrated that actual occurrence of these crops varied considerably, including maize being grown in an area previously considered ‘too dry’ for cultivation. Field sampling also revealed sporadic, and rare, plantings of sweet potato along the southern border of the scheme in plots not allocated or under the governance of the board. These findings have considerable impact, as the intended spatial arrangement of crop types by the farmer cooperative influences the water distribution throughout the scheme during the dry season.

Classification accuracies for each seasonal map image were assessed with confusion matrices created in ArcMap™ 10.5.1. Confusion matrices are a cross tabulation procedure that reflect the agreement between the produced land cover raster and ground truth [38]. Test pixels were evenly distributed across the study areas. The numbers of test pixels for each class were selected to ensure that at least 10-times the number of test pixels were selected per class as there were classes [39]. Four accuracy tests were applied for each classification. Kappa (κ) analysis is a discrete multivariate technique, commonly used for assessing classification accuracy [40]. The κ statistic is an estimation of the agreement between the classification map and reference (test pixels) data (see: [39]) where both the correctly classified and misclassified test pixels are considered. κ is computed as:

$$\hat{K} = \frac{{N\mathop \sum \nolimits_{i = 1}^{k} x_{ii} - \mathop \sum \nolimits_{i = 1}^{k} \left( {x_{i + } \times x_{ + i} } \right)}}{{N^{2} - \mathop \sum \nolimits_{i = 1}^{k} \left( {x_{i + } \times x_{ + i} } \right)}}$$

where $N$ is the total number of observations, $k$ is the number of land cover classes (e.g., rows) in the matrix, $x_{ii}$ is the number of observations in row $i$ and column $i$, and $x_{i + }$ and $x_{ + i}$ are the marginal totals for row $i$ and column $i$. Landis and Koch [41] consider values > 0.80 as strong agreement, values between 0.40 and 0.80 as moderate agreement, and < 0.40 as poor agreement. The κ coefficient value is 0.70 for the rainy season classification, an indication of satisfactory agreement between classified imagery and reality. The dry season κ coefficient value is 0.88 for the dry season, an indication of strong agreement. The overall accuracy is the product of the correctly classified test pixels in each class, divided by the total number of test pixels. The overall accuracy for the rainy season classification is 80%; the dry season classification is 93%. Results of classification accuracy assessments are summarized in Tables 1 and 2.

**Table 1:** Rainy season classification accuracy assessment

	Rice	Maize	Non-agricultural	Total	P.ac (%)	O.E. (%)
Rice	29	3	4	36	96.6	3.3
Maize	1	21	4	26	70	19.23
Non-agricultural	0	6	22	28	73.3	26.6
Total	30	30	30	90		
U.ac (%)	80.5	80.76	78.57			
C.O. (%)	19.4	19.23	21.42			
Overall accuracy	.80					
Kappa	.70					

*P.ac.* producer’s accuracy, *U.ac.* user accuracy, *E.O.* omission errors, *C.O.* commission errors

**Table 2:** Dry season classification accuracy assessment

	Fallow	Vegetated	Non-vegetated	Total	P.ac (%)	O.E. (%)
Fallow	38	0	4	42	95	5
Active agriculture	0	37	0	37	92.5	7.5
Bare earth	2	3	36	41	90	10
Total	40	40	40	120		
U.ac (%)	90.5	100	87.8			
C.O. (%)	9.5	0	12.2			
Overall accuracy	.93					
Kappa	.88					

*P.ac* producer’s accuracy, *U.ac* user accuracy, *E.O.* omission errors, *C.O.* commission errors

### Anthropogenic influence

#### BVIS structure and irrigation

**Fig. 7:**
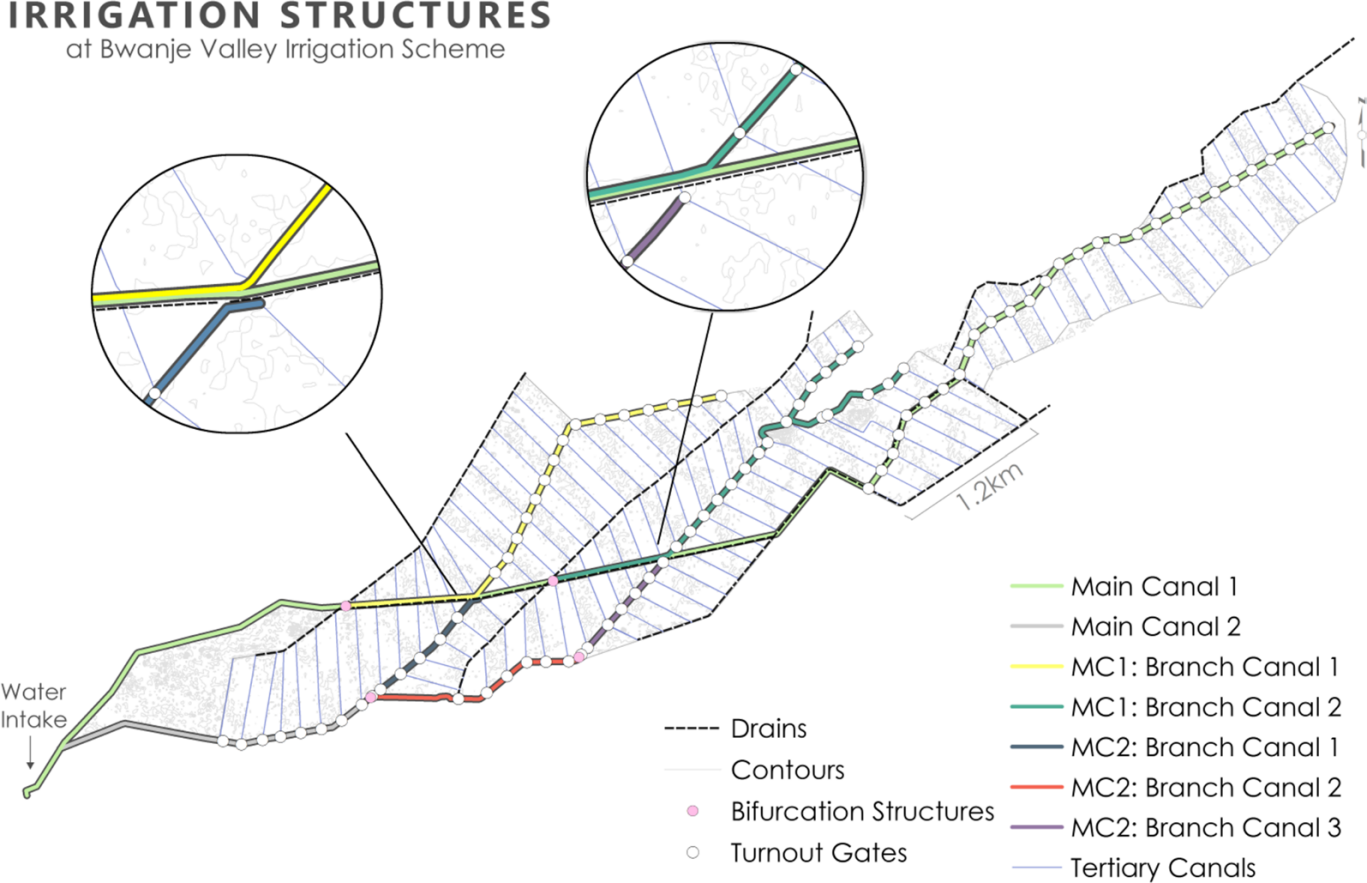
Irrigation engineering structures at BVIS including canals and water control structures

Crop distribution and growth in an irrigation scheme are heavily influenced by the scheme’s engineering. Mapping the irrigation structure at BVIS assisted in delineating between agricultural plots and agricultural infrastructure during classification and in understanding the movement and distribution of water across the landscape. Main and branching canals were mapped throughout the scheme along with the locations of the scheme’s bifurcation structures and division gates (Fig. 7). Division boxes are irrigation structures engineered to direct the flow of water between two or more canals through openings [42]. These openings are equipped with gates that at BVIS regulate the amount of water passing from branching to tertiary canals. Division gates were recorded at 105 points across the scheme and mark the beginning of each tertiary canal. Comprehensive technical descriptions of the engineering of BVIS are provided by JICA [30] and Kamwendo [43].

**Fig. 8:**
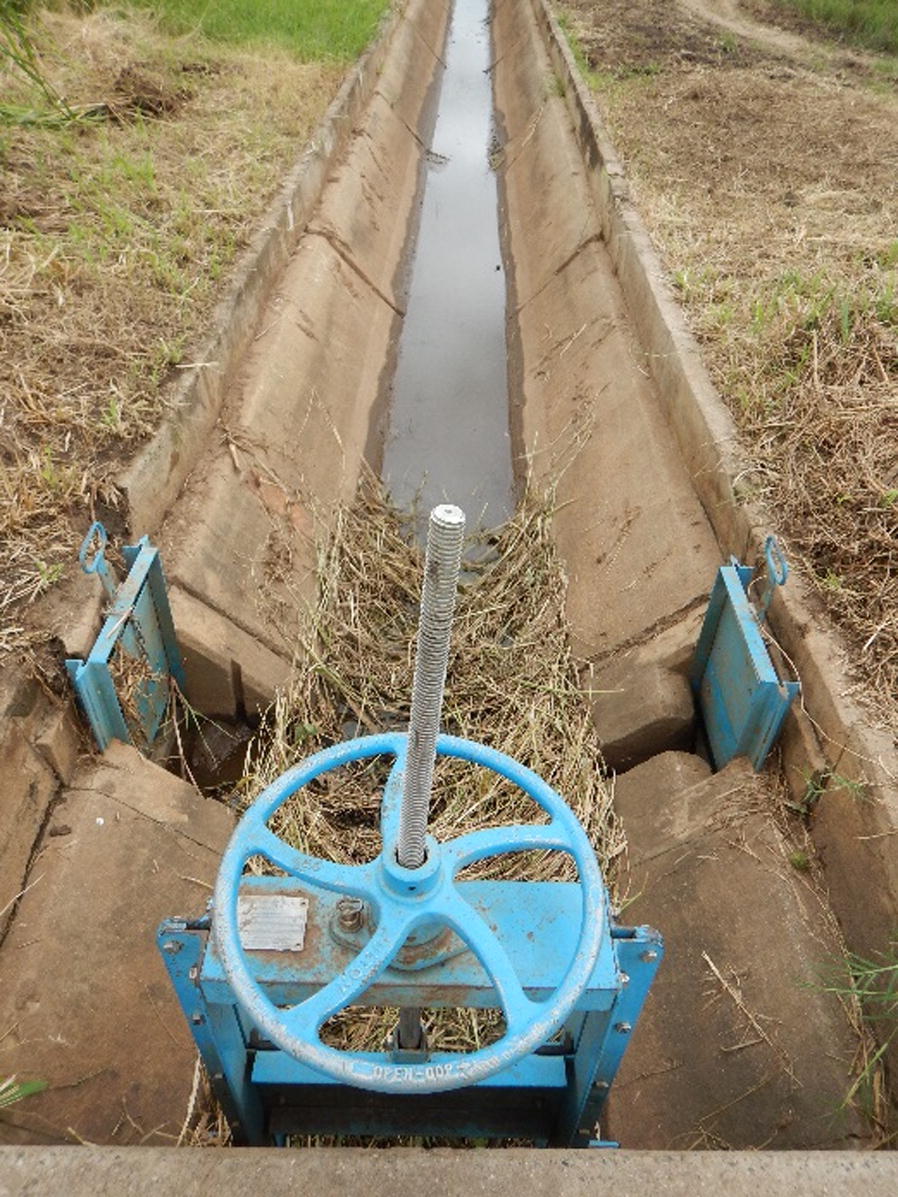
Mounded grass and debris placed in front of a branching canal slide gate at BVIS to prohibit water flow

Irrigation scheduling is the responsibility of the water use association (WUA) at BVIS [M. Tarsizio, pers. commun., 2016]. During the rainy season, scheme management practices a 3-day irrigation schedule; water is directed along one branch canal for 3 days, then redirected to another branch canal. No specific irrigation schedule is followed during the dry season; water is directed along branching canals based on the appearance of crop stress [M. Mafosha, pers. commun., 2016]. Previous research has highlighted conflict between scheme management and farmers on the basis of water regulation including the appointment of water guards, who control the allocation of water to each branch canal, and annual water fees [44]. Notable efforts to increase water access observed during field surveys included the placement of debris across control gates (Fig. 8). Further, two farmers were observed during sampling re-routing tertiary canal flow direction by removing silt preventing water access to their field, only to mound it further down the canal in order to halt the flow of water.

**Fig. 9:**
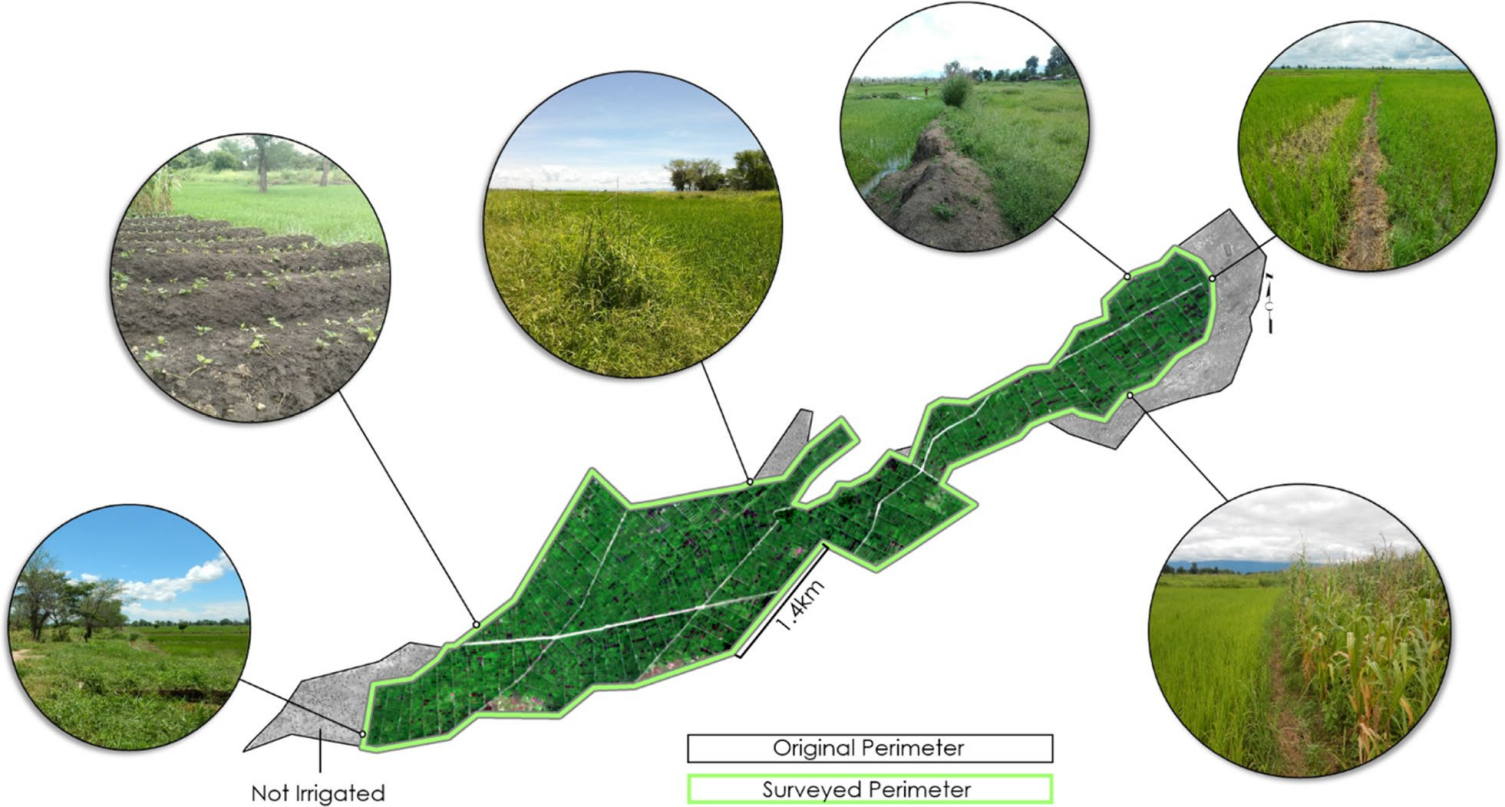
Original working perimeter (black) used for this study as georeferenced from JICA (2005). Surveyed perimeter (green) captured during 2017 field survey. Field photos depict variable land cover along the operational boundary of the scheme

Throughout the literature there are notable differences in how the perimeter of BVIS is mapped, particularly in the southeastern portion of the scheme (e.g. [30, 36, 44, 45]). Analysis was initially based off a perimeter georeferenced from [36] and verified through visual inspection of Google Earth Pro v.7.3.1 imagery. However, it became increasingly apparent during field surveys conducted in 2016 that this working perimeter did not accurately reflect the operational boundary of the scheme. Thus, a field survey delineating the operational boundary of the perimeter was conducted in March 2017. The perimeter was measured by walking the boundary of the scheme with GPS tracking enabled on a Garmin™ Monterra GPS device with points recorded at 3-s intervals (Fig. 9). To ensure the boundary’s positional accuracy, a member of BVIS management accompanied field researchers while performing the survey.

#### Drainage

**Fig. 10:**
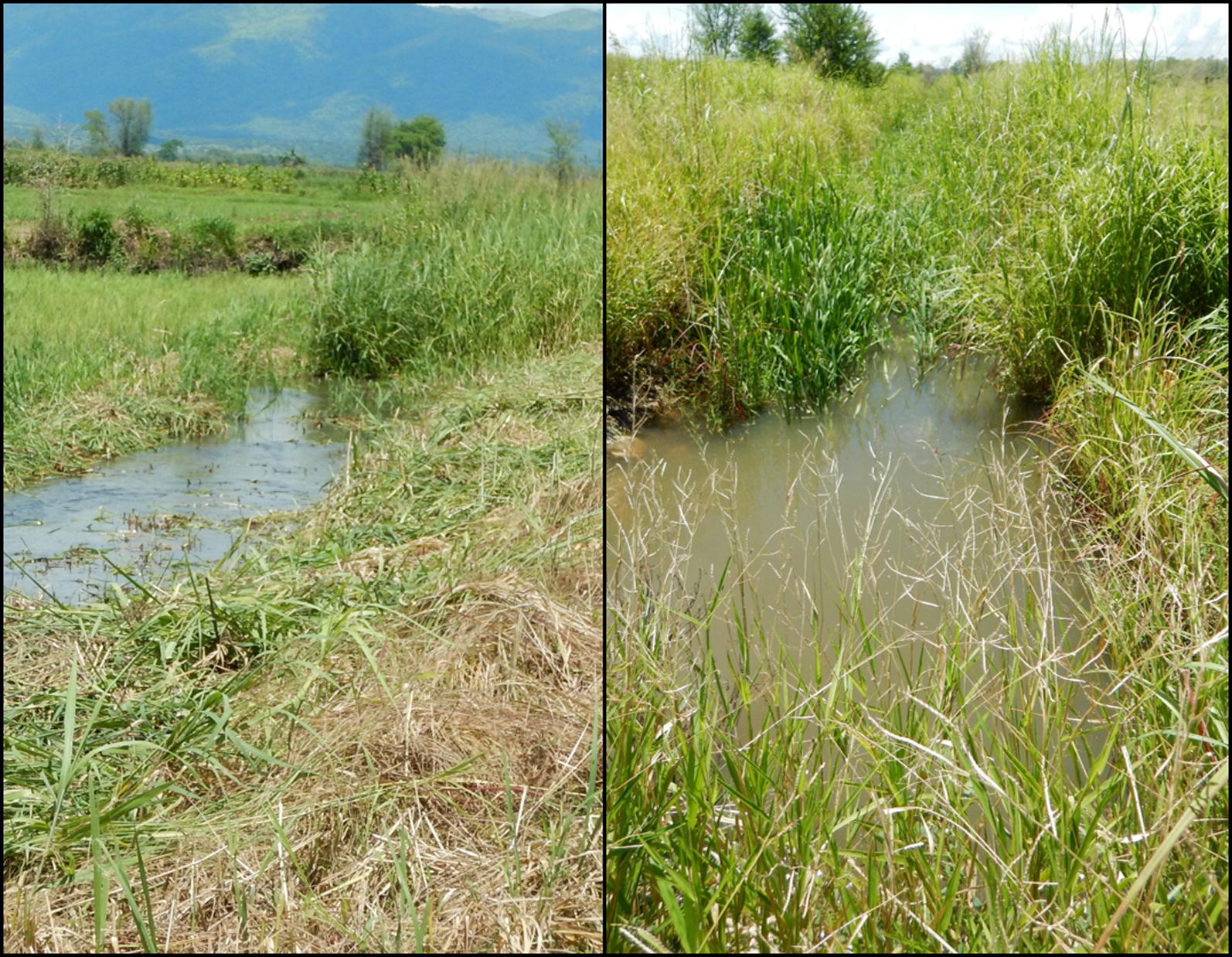
Evidence of improper maintenance of surface drains; the growth of tall grasses chokes drainage canals preventing proper redistribution of water resources across the scheme

While attempts were made to map the drainage system at BVIS during field surveys, many drainage canals were indiscernible as a result of either unmanaged grasses or rice plants allowed to grow within the drainage areas. As such, location of each of BVIS’s nine drains were georeferenced from the ‘Basic Design Study Report on the Project for the Rehabilitation of Bwanje Valley Irrigation System in the Republic of Malawi’ [36]. Surface drains facilitate the movement of excess water caused by either rainfall or the application of too much water [42]. Improperly functioning surface drains can lead to waterlogging, allowing for pooling of water at the soil surface [42]. Drainage at BVIS occurs in two distinct fashions: tail water is either collected, then redirected through a series of surface drains from one area of the scheme to another or is uncontrolled once leaving the tertiary canals and permitted to move naturally beyond the scheme’s boundaries. The scheme has four main drains that total 17.3-km [36]. These trapezoidal earth canals have a maximum allowable velocity of 0.75 m/s, with an allowable unit area of drainage discharge of 7.64 l/s/ha [36]. Drains at BVIS are susceptible to many of the same ongoing maintenance issues as those found in tertiary canals: portions of drains were choked with weeds and silt during surveying. De-silting and weed management of drains (and branching canals) are shared responsibilities among the farmers and WUA. Farmer names are written along a portion of the branching canal adjacent to their plot(s) at which point drain and branching canal maintenance for this area becomes their responsibility. Failure to maintain these areas may result in a fine from the WUA, though scheme management admits there is often a lack of enforcement resulting in untidy irrigation structures [M. Mafosha, pers. commun., 2019]. Figure 10 shows a section of a main drainage canal that has been cleared in the background. Yet, poor management practices on the part of the farmer operating the adjoining plot has allowed for the proliferation of tall grasses to grow. These grasses restrict water flow and redistribution of resources to other parts of the scheme. The variability of drainage maintenance has considerable influence on the area(s) of BVIS that favour mosquito breeding pool formation.

#### Management

At the onset of the scheme’s operation, BVIS was managed by the Malawi government, but by 2003 power dynamics shifted and farmers organized into a Cooperative. In 2004, the Cooperative was registered with the Ministry of Industry and Trade [45]. The capital to form the Cooperative was secured through a “One Village One Product” loan; a Japanese regional development program that aimed to develop one local product for trade on both domestic and local markets [45]. At BVIS, this product is rice. The BVIS Cooperative comprises farmers elected by scheme participants who serve on either the executive committee (8 members), or subcommittees (22 members). Subcommittees include: discipline, health, auditing, finance, marketing, and production. A scheme manager works in cooperation with the farmer cooperative to oversee daily scheme operation.

The types, quantities, and locations of agricultural crops grown at BVIS are governed by the farmer Cooperative. Farmers apply seasonally to grow specific crop types. Across the scheme, farmers practice monocropping; intercropping is discouraged [M. Mafosha, pers. commun., 2016]. Each season, farmers pay a water fee of Malawi Kwacha (MK) 1000 (~ 1.20USD) per ~ 30 × 30-m plot of land [M. Mafosha, pers. commun., 2019]. In addition, farmers are required to pay an annual MK5000 participation fee [M. Mafosha, pers. commun., 2019]. Farmers cultivate an average of five plots per season [M. Mafosha, pers. commun., 2019]. Though rare, farmers have the option of operating a plot ‘not allocated’ by the farmer Cooperative. In these types of arrangements, farmers are provided with a plot of land at the standard participation fee rate. However, they are not provided with water access in exchange for their ability to exercise governance over the crop type(s) under cultivation. In these situations, tertiary canal direction is rerouted to inhibit water access. If found to have tampered with the system so as to obtain water on an unallocated plot, farmers face a fine of MK5000 (~ $7 USD) [M. Tarsizio, pers. commun., 2016].

During dry season field surveys, very few unallocated plots were observed, and all were located along the southwestern border of the scheme adjacent to the Namikokwe River. The most obvious feature was the presence of intercropped maize and beans. It is these plot’s location in relation to the Namikokwe River that allows farmers to more easily cultivate them in an unallocated manner. Irrigation is conducted by watering can, a widely practiced method of irrigation for smallholder farmers [46]. While watering can irrigation is a simple and effective means of irrigating, carrying water is labour-intensive and regular watering is required, limiting areas that can be effectively irrigated.

#### Bwanje Dam

BVIS continues to evolve in an effort to meet food production demands. A considerable limitation to the success of BVIS is the availability of water resources, either rain fed or from the Namikokwe River. As intended, the Japanese estimated that BVIS could only support roughly 150 ha of dry season crops [28]. Though, Veldwisch et al. [28] report that the information that BVIS was not meant to supply water year-round came as a surprise to farmers during the first dry season. As a result, the Department of Irrigation sought to construct a dam in an effort to improve water availability and expand dry season cultivation at the scheme. Funded by the European Union [47], the project began in June 2016 [48]. The rock fill dam is the largest in the South African Development Community at 40 m high and approximately 150 m long with a storage capacity of 500 million cubic liters [47]. Construction was completed in October 2018 and the dam later launched in December 2018 [49]. The dam is expected to provide BVIS with sufficient water resources to cultivate 600 ha of rice during the dry season and additional horticultural crops (maize, common bean, cowpea, and soya). Future plans include expanding irrigable area at BVIS to 2000 ha [M. Mafosha, pers. commun., 2019].

#### Breeding pool suitability model construction

The spatial distribution of water bodies at BVIS is influenced by seasonality, soil properties, timing and intensity of irrigation, drainage, land cover, crop water requirements, and management. It would be misleading to present a single maximum estimate of breeding potential at BVIS given the variable nature of the factors influencing their spatio-temporal distribution. Rather, projected distributions are presented under three scenarios: rainy season, dry season with limited water resources, and a dry season with abundant water resources. For each scenario, projected distributions represent the seasonal peak period.

**Fig. 11:**
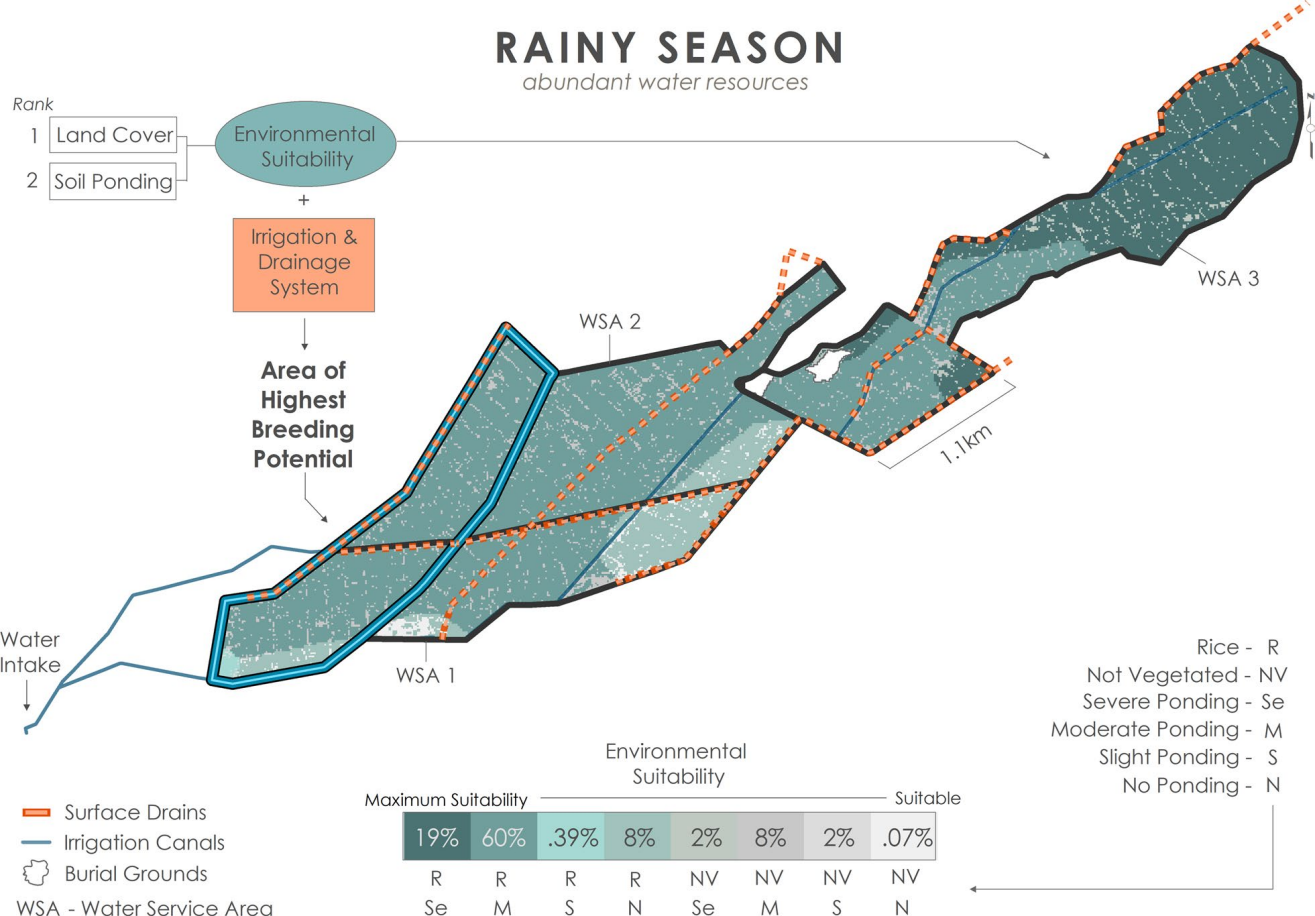
Breeding scenario under abundant water resource conditions during the rainy season

**Fig. 12:**
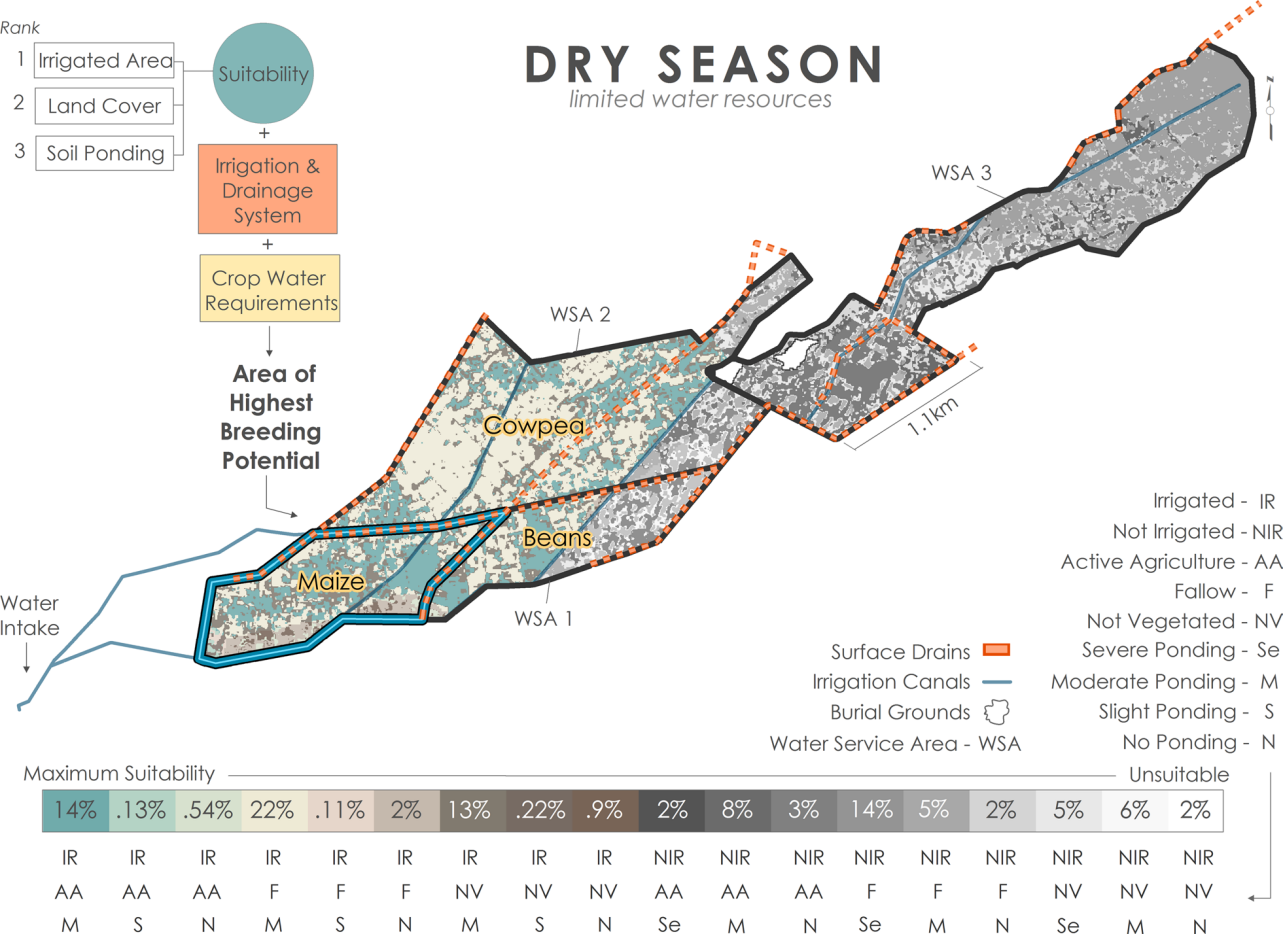
Breeding scenario under limited water resource conditions during the dry season

**Fig. 13:**
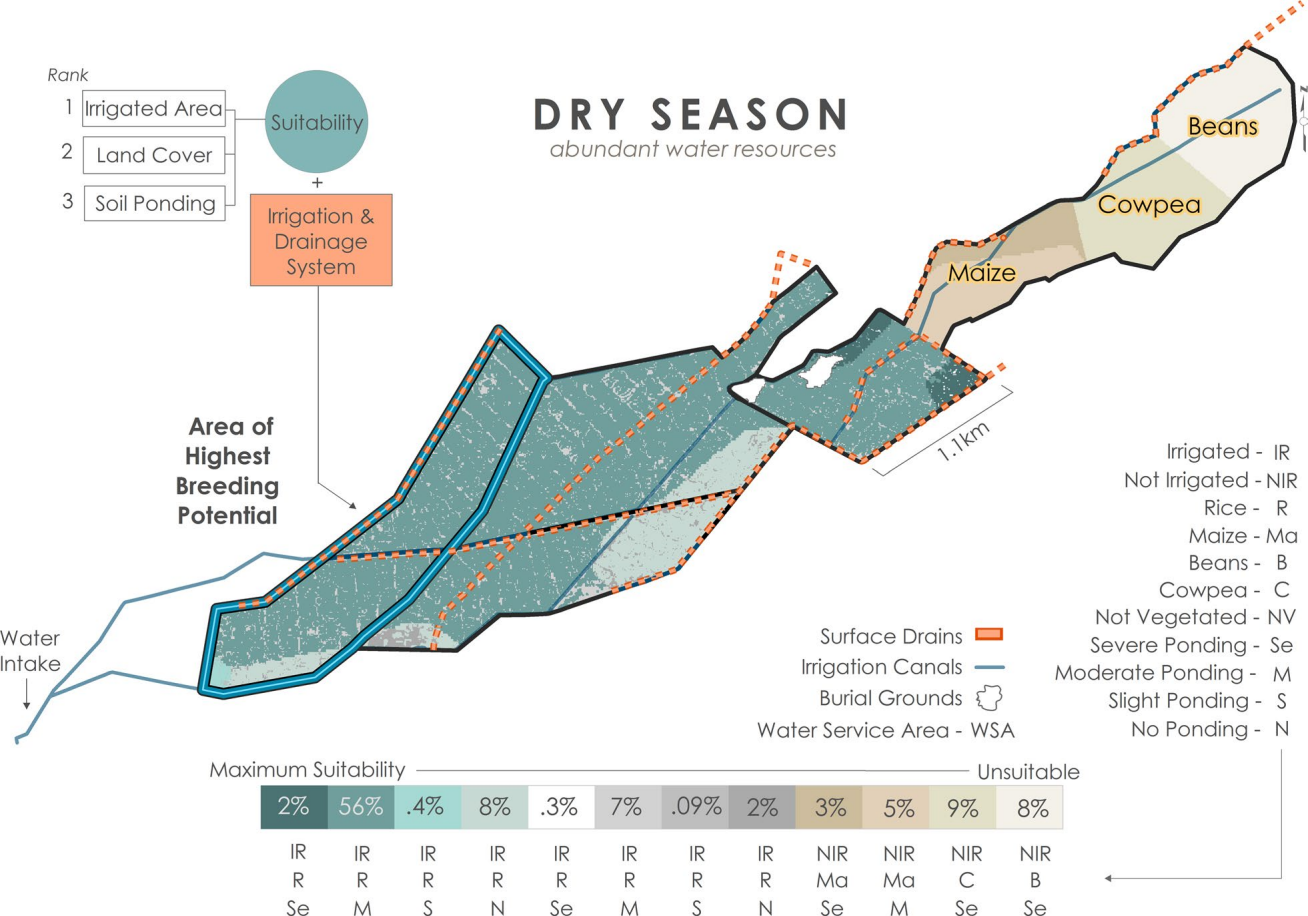
Breeding scenario under abundant water resource conditions during the dry season as a result of the construction of the Bwanje Dam

Breeding pool suitability was determined using a spatial multi-criteria model where suitability is defined as areas that facilitate the creation and persistence of mosquito breeding sites. Predictor maps for environmental variables (irrigated area, land cover, soil ponding potential) were created in ESRI ArcMap™ 10.5.1, then combined (i.e., overlayed) to create suitability classes of variable combinations. Suitability classes were ranked according to the combination of variable’s influence on the distribution of breeding pools at BVIS. Ranking of environmental variables was consistent for all scenarios: irrigated area, land cover, and soil type considered most to least influential, respectively. Irrigated area is binary, depicting irrigated (1) and non-irrigated (0) areas in the dry season. Information on irrigated area were provided during onsite interviews [M. Tarsizio, pers. commun., 2016]. It is assumed that insufficiency of water resources in areas that do not receive irrigation limits breeding opportunity in the absence of seasonal rains. The irrigated area variable is omitted from the rainy season model; all plots at BVIS are irrigated during the rainy season. Land cover types in irrigated areas, particularly in the dry season, are a strong indication of water supply received. In active agricultural areas, quantity and timing of irrigation reflect individual crop’s water requirements. It is prudent to note that agricultural crop types and their distribution are governed by the BVIS Cooperative. When water resources are limited, distinct spatial arrangements of crop types are valuable for irrigation planning and allocation of water resources. During the dry season irrigation scheduling is governed by the appearance of crop stress, a function of the crops fundamental water requirements. Crop types that require more water are irrigated more frequently than others. Maize and common bean require on average 500–800 mm and 300–500 mm per growing period, respectively [50]. Cowpeas are most often grown under dryland, not irrigated conditions given their ability to withstand drought conditions. Annual rainfall for geographical areas producing cowpeas averages 400–750 mm [51]. As such, it is assumed that breeding abundance is higher in irrigated spaces cultivating maize than common bean and cowpeas. Mosquito survival at BVIS is dependent on both the creation and persistence of breeding sites. Beyond where and to what extent water is applied at BVIS, soil drainage influences the spatio-temporal availability of surface water for mosquito breeding. Breeding opportunity will be higher in areas where soil’s ponding potential is severe in contrast to areas with better drainage. Each variable and their respective value’s ranking are provided in Table 3. Variable class combinations and the percent area they occupy at BVIS are available for each modeled scenario in Figs. 11, 12, 13.

**Table 3:**
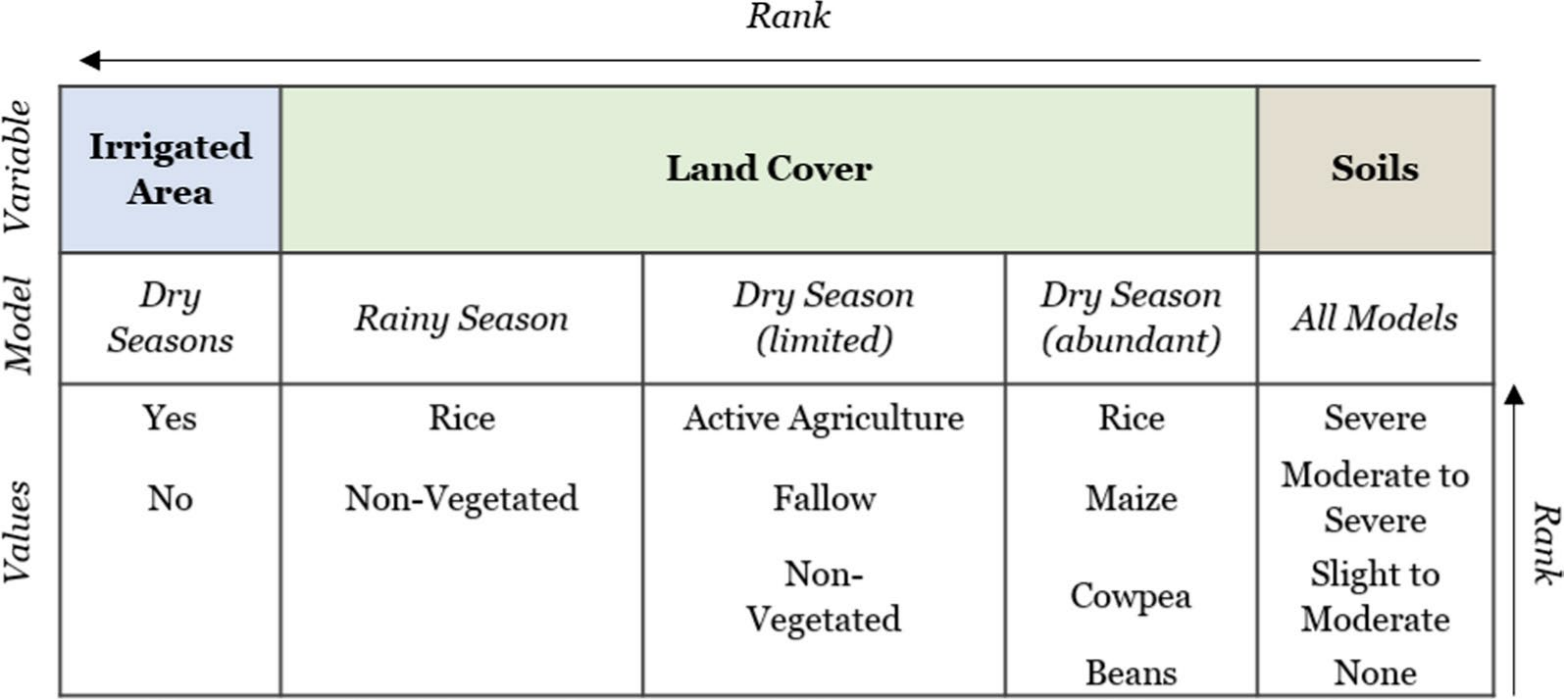
Ranking of variables and their values used in model construction

The distribution of breeding pool suitability in an irrigated scheme is a product of both environmental and anthropogenic factors. As such, for each scenario the outputs of the environmental sub-models described in the preceding paragraph are combined with information on the irrigation and drainage system at BVIS to elucidate areas of highest breeding risk. The influence of irrigation and drainage in all scenarios is approached from an understanding of the scheme’s irrigation engineering: the structure of the scheme is divided into three water service areas (WSA) based on the design of irrigation canals, their flow direction, and the location of drains across the scheme. Detailed information on anthropogenic influence on the distribution of water bodies is described in forthcoming sections for each scenario.

## Results

### Rainy season

Pervasive mosquito breeding occurs within plots across BVIS during the rainy season as a function of: (1) abundant water resources for rice cultivation both through precipitation and irrigation; (2) the soil’s susceptibility to ponding; and (3) regular irrigation following BVIS’ standard rainy season schedule. There are eight suitability classes ranging from suitable to maximally suitable for formation and persistence of mosquito breeding pools (Fig. 11). Seasonal rains render even non-vegetated areas including roadways regularly traversed by motorbikes and cattle suitable, though these areas contribution to the distribution of breeding sites is limited to only 12.07% of the total area. The largest percentage of land area (60%) is characterized by rice cultivation and soils with moderate ponding potential. Nineteen percent of BVIS is maximally suitable contrasted with only 0.07% of total area classified as suitable. Maximum suitability according to environmental variables occurs within the northeastern section of WSA3. Here the ponding potential based on soil ponding characteristics in combination with rice cultivation and regular flooding from irrigation and seasonal rains create an environment regularly conducive for mosquito breeding. Yet, environmental characteristics alone do not effectively describe water distribution and persistence as a function of irrigation management for irrigated schemes. In fact, field surveys showed that WSA3 receives less water than WSA1 and WSA2 by virtue of (1) its situation to the headworks (water intake) of the scheme and the necessary diversion of water upstream to branching canals; and (2) backlogging in WSA1 and WSA2 as a result of inefficient drainage and re-direction of tail water. Between irrigation application periods, drying out of plots situated furthest from the branching and main canals were observed in WSA3, which was a noted source of frustration among farmers whose plots were located in this area. However, the quantity and regular application of irrigation in combination with the inefficiency of tail water drainage leads to total inundation of rice plots in the western most portion of WSA1. As a result, this area exhibits the highest breeding potential during the rainy season (Fig. 11).

### Dry season: limited water resources

The opportunity for breeding during the dry season is directly influenced by water resource availability. This is evident in the reduction of cultivated land area from 800 ha in the rainy season to < 300 ha in the dry. Model results elucidate 18 suitability classes ranging from unsuitable to maximally suitable (Fig. 12). Fourteen percent (112 ha) of BVIS is maximally suitable during the dry season and characterized by irrigated, active agriculture occurring on soils with moderate ponding potential. Three classes of suitability summing to 13% of BVIS include non-irrigated, active agriculture. It is reasonable to assume that irrigation is being applied in some fashion (e.g., watering can or treadle pump) in these areas, however water application will be insufficient to permit the persistence of water bodies long enough for larvae to mature to adult stages vectors in these areas. The influence of drainage and redirection of tail water has less of an influence on the aggregation of water bodies as often water resources are limited to the point that entire tertiary canals are unable to be serviced with water; very little water is redistributed by drains. Crop types under cultivation are maize, common bean, and cowpeas. As a result, areas of greatest concentration of breeding potential are expected to be in the easternmost area of WSA1 based on the historical spatial arrangement of crop types dictated by the BVIS Cooperative (Fig. 12). This area is historically allocated to maize cultivation. In addition, its location nearest to the scheme’s headworks ensures that even despite limited water resources, irrigation water is supplied to these tertiary canals. This projection assumes that most farmers adhere to the cooperatives crop distribution guidelines.

### Dry season: abundant water resources

Given the significant hydrological changes expected at BVIS from the completion of the Bwanje Dam, in this scenario the impact to breeding pool distribution during the dry season where abundant water resources are present is considered, coupled with a primary change to rice agriculture. The projected 600 ha of irrigable area at BVIS will be those located closest to the scheme’s headworks. As such, rice cultivation is limited to these areas. The remaining 200 ha area is expected to be cultivated by horticultural crops given the presence of residual soil moisture and sloping topography owing to the gravity-fed design of the scheme. Irrigation scheduling will follow a 3-day schedule and drainage should function in a similar fashion to that of typical rainy seasons. Model results show twelve suitability classes ranging from unsuitable to maximally suitable (Fig. 13). Under these conditions, pervasive breeding is expected throughout the 600 ha area of irrigated rice plots. The largest concentration of breeding sites (56%) is predicted to occur in irrigated rice plots with moderately draining soils, characteristic of nearly 74% of the total irrigated area. Area of highest breeding potential is in the western portion of the scheme, as a function of water availability, ponding potential of soils, and inefficient drainage.

## Discussion

Risk potential for mosquito breeding at BVIS is seasonally asymmetrical across the scheme owing to environmental and anthropogenic factors. The three scenarios presented illustrate how perturbations to the irrigated system in the form of water availability, water management, and crop cover influence the distribution of aggregated water bodies. The noted association between irrigated agriculture and proliferation of mosquitoes is not novel [53–55]. However, treating irrigated areas as homogenous spatial units leads to inaccurate conclusions on breeding pool formation, persistence, and potential for malaria risk. It is one thing to assert that irrigation encourages mosquito production, it is quite another to answer where to expect breeding to occur. Particularly for irrigated areas that practice seasonal crop rotation, mixed cropping, or intercropping, the spatio-temporal distribution of crop cover can have profound impacts on the distribution of water resources across irrigated areas and, thereby, breeding potential.

Seasonal rains are critical to determining the spatial arrangement of water bodies at BVIS. Not only do the rains provide water necessary for oviposition indiscriminately across the scheme, but the amount of rainfall dictates water availability for irrigation supplied by the Namikokwe River. Not surprisingly, the rainy season model shows the highest percentage of suitable area for breeding overall (Fig. 11). However, it is not merely the amount of water supplied in an irrigated landscape that determines the aggregation of water bodies for mosquito breeding, but the management of water resources. In each scenario, timing and direction of irrigation along with inefficient drainage render the westernmost portion of BVIS the area of highest breeding opportunity. Results show that this area expands and contracts seasonally in response to water resource availability and management decisions (Figs. 11, 12, 13).

The expansion of irrigated agriculture is essential for mitigating food insecurity through increased crop production [56]. In Malawi, scaling irrigation has and continues to occur against the backdrop of numerous national policy frameworks (e.g., VISION 2020, Malawi Growth and Development Strategy I—III, Agricultural Sector Wide Approach I&II, National Irrigation Policy, Green Belt Initiative) often tied to strategies to increase agricultural productivity as a means of poverty reduction and economic growth. The current Irrigation Master Plan for Malawi intends to increase irrigable area from 104,298 to 220,000 ha by 2035 [15], including expanding irrigable area at BVIS from water resources provided by the Bwanje dam. Rather than limiting breeding opportunity as a function of water resources, the availability of continuous irrigation from the Bwanje Dam has the potential to change breeding distribution from that expected during a prototypical dry season, to an expected distribution more typical of rainy seasons. The consequences could be severe. Where breeding is restricted to less than 50% of the overall scheme area during a typical dry season (Fig. 12), the availability of water resources from the Bwanje Dam increases the temporal breeding landscape (Fig. 13), changing not only the distribution of breeding but potential seasonal variation in malaria transmission for those residing in close proximity to BVIS. It is prudent to note that increases in mosquito populations do not necessarily increase malaria risk. There are myriad factors that affect malaria transmission including the stability of malaria transmission in areas where irrigation is introduced [21], housing quality, availability of anti-malarial drugs [57], economic status [58], and the use of insecticide-treated bed nets [59].

**Fig. 14:**
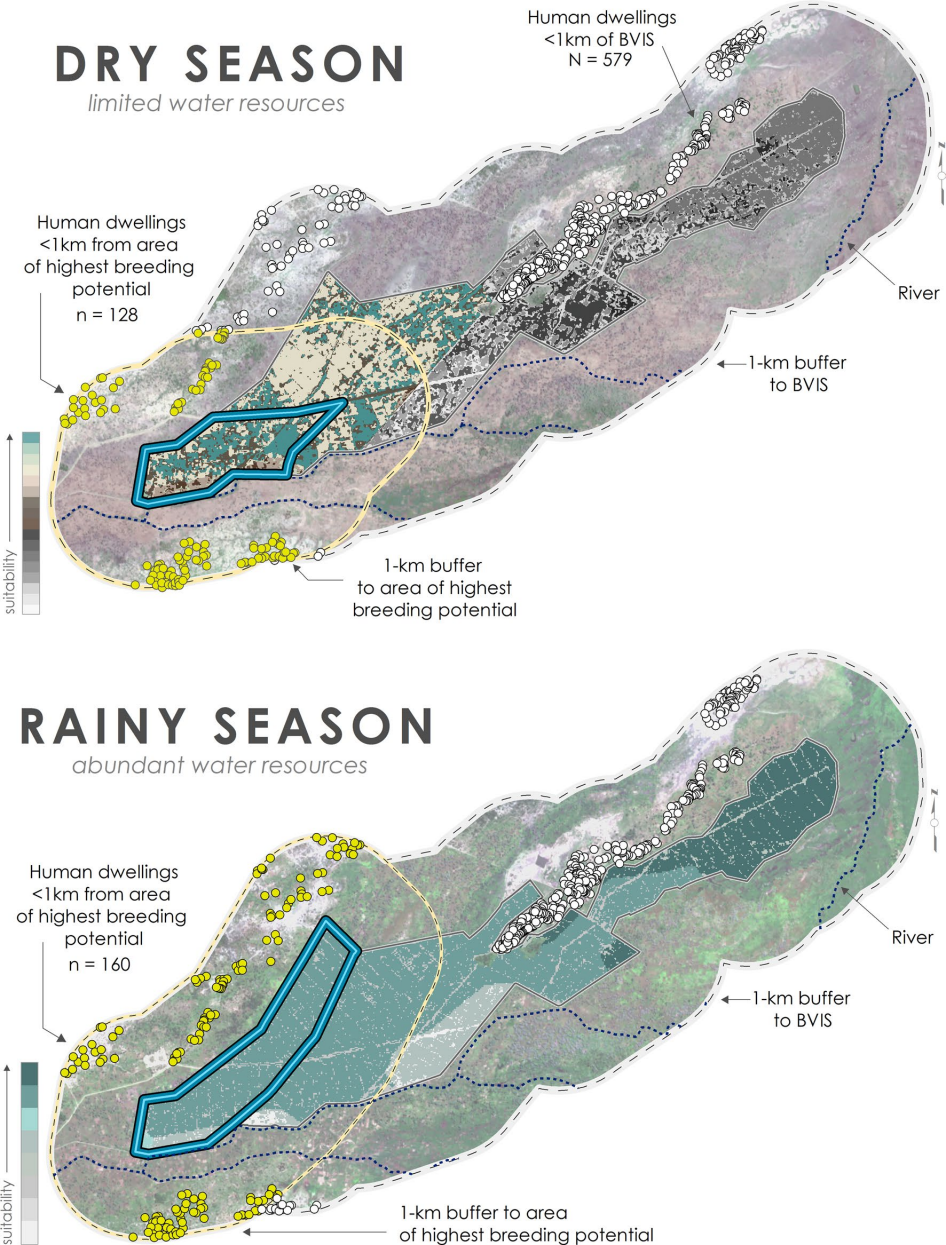
Seasonal variation in malaria vulnerability for populations residing within 1-km of BVIS. Human dwellings within 1-km of BVIS are depicted in white; dwellings within 1-km of predicted area of highest breeding risk are shown in yellow. Populations residing within 1-km of highest breeding risk are most vulnerable to malaria transmission

Principal malaria vectors in Malawi are *Anopheles gambiae* sensu stricto (s.s.), *Anopheles arabiensis*, and *Anopheles funestus* [60]; *An. gambiae* s.s. is particularly efficient at transmitting malaria [61]. As such, characteristics for *An. gambiae* s.s. are considered in the following analysis. To evaluate the potential impact of BVIS on malaria transmission for the surrounding area, human dwellings < 1-km of BVIS were identified using Google Earth Pro 7.3.2.5776 (Fig. 14). Constantini et al. [62] demonstrated that maximum flight distance for *An.* *gambiae* s.s. was > 1-km while Thomas et al. [63] reported 1.7-km; the conservative measure was adopted here.

A total of 579 human dwellings were identified within 1-km of BVIS. Temporal malaria risk will differ for populations living in these dwellings in contrast to those residing further away as a product of regular, surface water availability at BVIS for female mosquitoes to lay their eggs and larvae to survive through to adult stage. For anthropophilic mosquitoes, including members of the *Anopheles* *gambiae* complex, mosquito dispersal is short in areas of high human population densities [64]; mosquitoes will fly no further than necessary for a blood meal. It is expected that in human dwellings < 1-km of BVIS, the density of adult *Anopheles* mosquitoes will be greater than in dwellings further from the scheme as females seek out human dwellings for blood feeding and resting sites. This relationship will decline exponentially with distance from irrigated areas.

Results of this study have demonstrated that breeding risk is seasonally asymmetrical across BVIS, subsequently changing the geography of malaria risk for the surrounding area. Human dwellings located within 1-km of the predicted area of highest breeding risk at BVIS are most vulnerable to malaria transmission. In the dry season when water resources are limited, 128 human dwellings are located within this area. In the rainy season, the number of dwellings increases to 160; these households are also at risk during the dry season when water resources are abundant through provision via the Bwanje Dam. There are 105 dwellings in common between the rainy and dry seasons.

In estimating their probable locations, these models have limitations that promote relative breeding potential. First, there are no existing measurements for the amount of water at BVIS either diverted from Namikokwe, passing through branching or tertiary canals, or ultimately that passes into farmer's fields. Precise measurements on the total amount of water being applied to each field, in combination with information on irrigation scheduling and crop types would assist in developing models that estimate breeding potential as a function of total water volume and estimated root water uptake. These data could be coupled with local weather information to further refine estimations of water loss through evaporation. A related point is the absence of specific crop variety information at BVIS. This information would assist in better characterizing the scheme in relationship to the growing periods and specific root water uptake characteristics of crop varieties.

An added consideration to both the rainy season and dry season, abundant water resources scenario is the effect of stage of rice growth; non-consecutive planting times will affect the spatio-temporal distribution of larva species. *An. arabiensis* preferentially breed in open, sun lit pools [65]; characteristic of plots in preparation for, or just after transplanting. As rice plants begin to grow and water surfaces are shaded by vegetation, abundance of *An. arabiensis* declines [65]. *Anopheles funestus* s.s., however prefers breeding in areas with emergent vegetation and large, permanent or semi-permanent fresh water bodies [65]. Marrama et al. [66] showed that later, grain head formation and maturation stages of rice growth are associated with *An. funestus* breeding. Diuk-Wasser et al. [67] demonstrated a significant relationship between land use, including stages of rice, and abundance of *An. gambiae*. Vectorial capacities of mosquito species are not uniform. Because vector abundance is a critical factor to malaria transmission, cultivation practices will influence the disease ecology of the local area.

Seasonal peaks in malaria transmission most often occur during the late rainy season or immediately after its conclusion [25]. Dry season malaria transmission is limited by the reduction of inundated areas. During the dry season, optimal breeding conditions are expected in irrigated spaces given the presence of either formal or informal irrigation management measures. As a result, malaria transmission for those living within 1-km of irrigated spaces, including BVIS will not experience the same level of decline in transmission as those beyond 1-km from irrigated schemes.

## Conclusions

Small-holder irrigated schemes are not homogeneous spatio-temporal units, and breeding potential for mosquitoes varies in both space and time. Changes to the geography of breeding potential across irrigated spaces can considerably alter malaria risk and impact those living in close proximity. At present, food insecurity is driving the expansion of irrigated agriculture in many malaria endemic countries. This study demonstrates a generalizable approach that considers the environmental and anthropogenic factors that contribute to breeding risk in irrigated schemes, in turn providing an opportunity to examine potential changes to the spatio-temporal distribution of malaria transmission for surrounding areas. This model will assist with developing spatially and temporally targeted malaria management strategies for areas situated in close proximity to irrigated agriculture in an effort to continue increasing crop production without exacerbating malaria risk.

## Data Availability

The datasets generated and/or analysed during the current study are available in the Harvard Dataverse repository: 10.7910/DVN/9BZU2C, 10.7910/DVN/PZEJWA, 10.7910/DVN/QKFEVM.

## Abbreviations

BVIS: Bwanje Valley Irrigation Scheme
DEM: Digital Elevation Model
GBI: Green Belt Initiative
GoM: Government of Malawi
GPS: Global Positioning System
JICA: Japanese International Cooperative Agency
LULC: Land Use and Land Cover
LULCC: Land Use and Land Cover Change
MK: Malawi Kwacha
NIP: National Irrigation Policy
NDVI: Normalized Difference Vegetation Index
SRTM: Shuttle Radar Topography Mission
SSA: sub-Saharan Africa
WSA: Water Service Area
WUA: Water Use Association
